# Dosimetric Feasibility of Dose Escalation Using SBRT Boost for Stage III Non-Small Cell Lung Cancer

**DOI:** 10.3389/fonc.2012.00124

**Published:** 2012-09-26

**Authors:** Jaroslaw T. Hepel, Justin Peter, Jessica R. Hiatt, Salil Patel, Oluwademilade Osibanjo, Howard Safran, Bruce Curran, Thomas DiPetrillo

**Affiliations:** ^1^Department of Radiation Oncology, Rhode Island Hospital, Brown UniversityProvidence, RI, USA; ^2^Department of Hematology and Oncology, Rhode Island Hospital, Brown UniversityProvidence, RI, USA

**Keywords:** NSCLCa, SBRT, SAbR

## Abstract

**Purpose:** Standard chemoradiation therapy for stage III non-small cell lung cancer (NSCLCa) results in suboptimal outcomes with a high rate of local failure and poor overall survival. We hypothesize that dose escalation using stereotactic body radiotherapy (SBRT) boost could improve upon these results. We present here a study evaluating the dosimetric feasibility of such an approach. **Methods:** Anonymized CT data sets from five randomly selected patients with stage III NSCLCa undergoing definitive chemoradiation therapy in our department with disease volumes appropriate for SBRT boost were selected. Three-dimensional conformal radiation therapy (3D-CRT) plans to 50.4 Gy in 28 fractions were generated follow by SBRT plans to two dose levels, 16 Gy in two fractions and 28 Gy in two fractions. SBRT plans and total composite (3D-CRT and SBRT) were optimized and evaluated for target coverage and dose to critical structures; lung, esophagus, cord, and heart. **Results:** All five plans met predetermined target coverage and normal tissue dose constraints. PTV V95 was equal to or greater than 95% in all cases. The cumulative lung V20 and V5 of the combined 3D-CRT and SBRT plans were less than or equal to 30 and 55%, respectively. The 5 cc esophageal dose was less than 12 Gy for all low and high dose SBRT plans. The cumulative dose to the esophagus was also acceptable with less than 10% of the esophagus receiving doses in excess of 50 Gy. The cumulative spinal cord dose was less than 33 Gy and heart V25 was less than 5%. **Conclusion:** The combination of chemoradiation to 50.4 Gy followed by SBRT boost to gross disease at the primary tumor and involved regional lymph nodes is feasible with respect to normal tissue dose constraints in this dosimetric pilot study. A phase I/II trial to evaluate the clinical safety and efficacy of this approach is being undertaken.

## INTRODUCTION

Lung cancer remains the leading cause of death for both men and women in the US (Siegel et al., 2012). Although the results of treatment for early stage non-small cell lung cancer (NSCLCa) are encouraging, as many as 40% of patients present with stage III disease (Chang et al., 2008). The standard treatment approach for these patients is concurrent chemoradiation therapy with or without surgery. However, overall survival with this approach is expected to be only 15–35% at 5 years (Edge et al., 2010). Patterns of failure show that both local and systemic relapse are common. Local failure rate of 35–50% is expected with radiation therapy using standard fractionation (1.8–2 Gy/fx) and doses (60–70 Gy; Curran et al., 2011). In an attempt to improve upon local control and outcomes, radiation dose escalation has been investigated (Hayman et al., 2001; Narayan et al., 2004; Bradley et al., 2005,^2010^; Kong et al., 2005; Partridge et al., 2011). Stereotactic body radiation therapy (SBRT) is a highly precise and conformal method of delivering high doses of radiation therapy. SBRT has shown impressive local control rates of 85–95% in early stage lung cancer (Onishi et al., 2004,^2007^; Nagata et al., 2005; Nguyen et al., 2008; Baumann et al., 2009; Fakiris et al., 2009; Timmerman et al.Timmerman et al., 2010). Such improvement in local control has been correlated to the increased biologic equivalent dose (BED) achieved with the radiation dose schema of SBRT. In this study, we evaluated the dosimetric feasibility of using SBRT as a boost to gross disease (primary lung tumor and bulky mediastinal lymphadenopathy) following standard fractionated three-dimensional conformal radiation therapy (3D-CRT) to 50.4 Gy.

## METHODS

Anonymized CT data sets from five randomly selected patients with stage III NSCLCa undergoing definitive chemoradiation therapy in our department were selected. Selected patients had to have gross disease volumes amenable to SBRT boost. This was defined as primary tumor volume of less than or equal to 120 cc and hilar/mediastinal disease involving one to two nodal stations with volume less than or equal to 60 cc.

### 3D-CRT PLANS

Patients underwent CT simulation in the supine position with arms up using an arm shuttle and alpha cradle for immobilization. Standard CT-based 3D-CRT treatment plans using a three to five coplanar beam arrangement were generated to target the primary tumor and nodal GTV as defined by diagnostic PET and CT scans. A margin of 2 cm was placed around all gross disease to account for subclinical disease, set up error, and respiratory motion. Elective nodal irradiation was not performed. Dose calculation were performed using Pinnacle version 8.0m (Philips, Madison, WI, USA). Heterogeneity corrections were employed to correct for differences in tissue density; the collapsed cone convolution (CCC) method was used for dose calculation. A dose of 50.4 Gy in 1.8 Gy/fx was prescribed. Isodose plans and dose volume histograms (DVHs) were generated.

### SBRT PLANS

Target volumes for SBRT boost consisted of gross tumor only (GTV) with the standard PTV margin. Typically, patients undergoing SBRT in our department are simulated with appropriate immobilization using the BlueBAG BodyFIX system (Elekta, Stockholm, Sweden) and abdominal compression to reduce respiratory excursion. During simulation, respiratory gated image acquisition is performed to determine target tumor motion in order to generate a patient-specific ITV. Because patients were simulated for 3D-CRT treatment without SBRT appropriate immobilization and a respiratory dampening device, an accurate SBRT ITV was not available for these patients. A margin expansion of 1 cm in the superior and inferior directions and 0.5 cm in the radial directions was, therefore, used. This margin corresponds well to the typical margin expansion using for ITV and PTV in our SBRT patient population as well as what is reported in the literature (Fakiris et al., 2009). Dose calculation were performed using Eclipse version 8.6 (Varian, Las Vegas, NV, USA). Heterogeneity correction was used to correct for differences in tissue density and the anisotropic analytical algorithm (AAA) was employed for dose calculation. The primary lung tumor and the hilar/mediastinal nodal disease were planned separately. Intensity modulated radiation therapy (IMRT) was used employing an 8–14 non-coplanar beam arrangement. A composite of the two SBRT plans was generated to evaluate for any significant dose overlap and to calculate the volume of low dose spillage. Two prescription doses were evaluated: 16 Gy in two fractions and 28 Gy in two fractions. These doses were selected based on a planned dose escalation study with 16 Gy representing the starting dose and 28 Gy representing the maximum target dose. Plans were normalized such that 95% of the PTV was covered by 95% of the prescription dose.

### COMPOSITE PLANS

Composite plans between the 50.4 Gy 3D-CRT plan and SBRT boost plan were generated for the five patients using the fusion algorithm of Velocity version 2.5 (Atlanta, GA, USA). Composite DVHs and isodose plans were generated. Plans were optimized to meet dose constraints detailed in **Tables 1** and **2**. These dose constraints were selected based on commonly used and known dose volume constraints for these organs based on conventional fractionated and SBRT treatments. Cumulative dose limits between the 3D-CRT and SBRT plans were based on BED conversion to take into account differences in biological effect of varying dose per fraction. BED and equivalent dose in 2 Gy fractions (EQD2) calculation were based on the following equations.

$$\begin{array}{c} BED=n\times d\times [1+d/(\alpha /\beta )]andEQD2=n\times d\times (d+\alpha /\beta )/(2+\alpha /\beta ) \end{array}$$

**Table 1:** Stereotactic body radiotherapy boost dose constraints.

Organ	Volume	Dose	Endpoint (>/Grade 3)
Spinal cord	Max point dose	9 Gy (4.5 Gy x 2)	Myelitis
Lung	10%	7 Gy (3.5 Gy x 2)	Pneumonitis
Esophagus	<5 cc	12 Gy (6 Gy x 2)	Stenosis/fistula
Heart	<15cc	8 Gy (4 Gy x 2)	Pericarditis
Great vessels	<10 cc	24 Gy (12 Gy x 2)	Aneurysm
Brachial plexus	Max point dose	8 Gy (4 Gy x 2)	Neuropathy
Rib	<1 cc	16 Gy (8 Gy x 2)	Pain or fracture
Skin	<10 cc	16 Gy (8 Gy x 2)	Ulceration
Stomach	<10cc	8 Gy (4 Gy x 2)	Ulceration/fistula

**Table 2:** Cumulative dose constraints (EBRT and SBRT boost).

Organ	Volume	EQD2*	Endpoint (>/Grade 3)
Spinal cord	Max point dose	45 Gy	Myelitis
Lung	<35%	20 Gy	Pneumonitis
Lung	<65%	5 Gy	Pneumonitis
Esophagus	10 cm	60 Gy	Stenosis/fistula
Heart	25%	40 Gy	Pericarditis
Brachial plexus	Max point dose	66 Gy	Neuropathy

* With α/β of 3 used for late normal tissue effects except for spinal cord and brachial plexus were an α/β of 2 is used.

An α/β of 10, 3, and 2 were used for tumor control, late tissue affects, and late affects on spinal cord and neural structures, respectively.

## RESULTS

**FIGURE 1:**
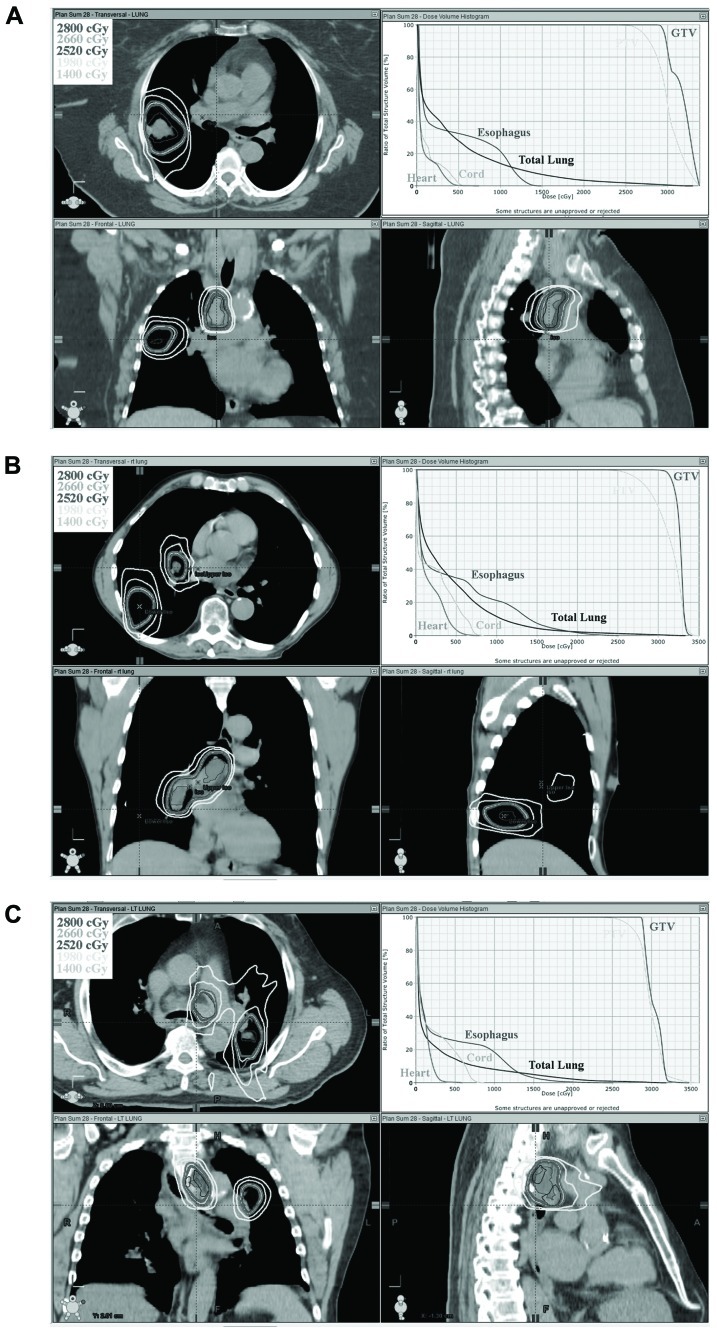
**Orthogonal isodose distribution and DVH of SBRT boost plan to 28 Gy in two fractions.** Three separate cases are depicted in **(A–C)**.

All five plans met predetermined target coverage and normal tissue dose constraints as defined in **Tables 1** and **2**. PTV V95 was equal to or greater than 95% in all cases. For the five cases studied, the cumulative lung V20 of the combined 3D-CRT and SBRT plans were 17, 30, 28, 33, 19 and 18, 30, 29, 34, 20% for low dose and high dose SBRT, respectively. The cumulative lung V5 was similarly within acceptable limits, less than 55% for all plans. The 5 cc esophageal dose was less than 12 Gy for all low and high dose SBRT plans. The cumulative dose to the esophagus was also low with less than 18% of the esophagus receiving doses in excess of 50 Gy. In addition, heart and spinal cord doses were low. **Table 3** shows target and normal tissue doses for 3D-CRT plans, low dose SBRT and high dose SBRT plans, and composite plans. The isodose distribution and DVH for each high dose SBRT boost plan is depicted in **Figure 1**.

**Table 3:** Dose statistic for 3D-CRT, SBRT, and composite plans.

Plan	GTVV100	Boost PTV	Max dose	Lung V5	Lung V10	Lung V20	Spinal cord	Esophagus	Esophagus	Heart V5	Heart V25
	(%)	V95 (%)	(%)	(%)	(%)	(%)	max dose (Gy)	V50 (%)	5 cc (Gy)	(%)	(%)
Case 1
3D-CRT	99	n/a	117	38	33	28	274	0	35.0	10	1
SBRT 16 Gy	100	97	121	16	5	0	3.3	0	5.2	0	0
Composite (16 Gy)	100	97	113	44	36	30	30.7	5	40.2	11	1
SBRT 28 Gy	100	97	121	28	14	3	5.7	0	8.9	0	0
Composite (28 Gy)	100	97	113	44	36	30	32.1	7	43.9	32	1
Case 2
3D-CRT	99	n/a	119	44	35	25	26.0	0	20.5	22	4
SBRT 16 Gy	100	97	125	15	3	0	4.8	0	6.8	0	0
Composite (16 Gy)	100	97	118	49	39	28	28.6	1	27.3	23	4
SBRT 28 Gy	100	97	125	31	12	2	8.4	0	11.9	3	0
Composite (28 Gy)	100	97	118	53	41	29	31.8	4	32.4	28	4
Case 3
3D-CRT	100	n/a	112	40	28	16	23.5	3	42.5	11	1
SBRT 16 Gy	100	97	129	9	3	0	5.0	0	6.6	0	0
Composite (16 Gy)	100	95	113	42	30	17	277	5	49.1	13	1
SBRT 28 Gy	100	97	129	14	8	2	8.7	0	11.5	0	0
Composite (28 Gy)	100	95	113	44	31	18	31.2	6	53.0	15	1
Case 4
3D-CRT	100	n/a	113	44	35	27.5	14.7	0	38.3	89	36
SBRT 16 Gy	100	95	124	10	2	0	4.1	0	4.7	0	0
Composite (16 Gy)	100	95	112.5	54	42	33	17.3	0.5	43.1	89	36
SBRT 28 Gy	100	95	124	33	8	1.4	7.2	0	8.1	5	0
Composite (28 Gy)	100	99	114	61	46	34	19.3	1.9	46.5	93.1	36
Case 5
3D-CRT	100	n/a	117	38	28	15	31.4	12.7	50.4	46.5	18
SBRT 16 Gy	100	95	117.7	6.2	1.3	0	5.3	0	5.3	14.1	0
Composite (16 Gy)	100	95	112	41.1	31.2	19	36.9	17.1	55.9	60.6	18
SBRT 28 Gy	100	95	117	12	4.7	0.8	9.3	0	9.4	20	0
Composite (28 Gy)	100	95	112	42.3	31.8	20	40	17.8	60.1	66.5	18

## DISCUSSION

The results of definitive treatment for stage III NSCLCa are disappointing with an expected 5 year survival of only 15–35% (Edge et al., 2010). RTOG 9410 help establish concurrent chemoradiation as the current standard treatment approach for these patients (Curran et al., 2011). Patterns of failure from this study have been reported and show that local-regional control with cisplatin-based chemotherapy and concurrent radiation therapy to 63 Gy in standard fractionation was only 65%. Furthermore, 25% of patients experience in-field local failure only without distant metastasis (Curran et al., 2011). The addition of surgery to improve upon suboptimal local control was evaluated in INT/RTOG 0139 (Albain et al., 2009). This trial showed improved 5 year progression-free survival from 11 to 22%, but overall survival was not improved. The lack of overall survival benefit has been attributed to early treatment related morbidity and mortality of the tri-modality arm particularly in patients who required pneumonectomy. Many high volume and academic centers still favor a tri-modality approach for select patients based on evidence that the morbidity and mortality rates at these centers is low with this approach (Allen et al., 2008). Nonetheless, many patients are not ideal candidates for surgery due to concurrent cardiac and/or pulmonary disease or due to the requirement for pneumonectomy. Attempts to improve upon local disease control with radiation dose escalation have thus been undertaken. This approach is grounded soundly in the fundamental principles of radiation biology. The higher the radiation dose, the larger the fractional cell kill, and hence the greater the probability of disease control. Kong et al. (2005) reported on the results of a dose escalation trial which showed improved overall survival with high radiation doses. At 5 years, overall survival was 4, 22, and 28% for patients receiving 63–69, 74–84, and 92–103 Gy, respectively. Hayman et al. (2001) reported on dose escalation from 63 to 102.9 Gy with patients stratified based on treatment volumes. Reported 3 year survival was 18%. RTOG 9311 showed feasibility of dose escalation to 83.8 Gy (Bradley et al., 2005). However, with concurrent chemotherapy, a maximum tolerated dose of only 74 Gy could be achieved in RTOG 0117 (Bradley et al., 2010). Partridge et al. (2011) performed a systematic review and modeling analysis of published trials of dose escalation. They found a clear dose–response relationship for improved disease-free survival. Interestingly, the best outcomes were seen in hypofractionated regimens.

RTOG 0617/CALGB 30609, a phase III randomized trial, was designed to evaluate the efficacy of dose escalation using concurrent chemoradiation for stage III NSCLCa (Bradley et al., 2011). This trial consists of a 2 by 2 factorial design comparing 60 vs. 74 Gy with and without cetuximab. This trial unexpectedly was closed early for futility after enrolling 450 patients. No difference in the primary endpoint of overall survival was seen between the high and low dose arms. Several explanations can be postulated for why this study failed to show a benefit from dose escalation. First, failure to control systemic disease may be overshadowing any benefit from improvements in local disease control. Second, toxicity from dose escalation using conventional 3D-CRT may limit any benefits gained. Third, 74 Gy is not a high enough dose to result in a high rate of local disease control. And lastly, accelerated tumor repopulation that typically begins after 4 weeks of treatment may be mitigating any tumor cell kill gained from dose escalation using a protracted standard fractionated regime.

The rationale to use SBRT boost as a method of dose escalation addresses the later three postulates. The conformal nature of an SBRT approach limits the amount of normal tissues receiving radiation thereby allowing the potential for safer radiation dose escalation. By delivering the boost in just two fractions, the detrimental effect of accelerated tumor repopulation is eliminated. And finally, the dose of radiation can be significantly elevated using an SBRT approach. Although the optimal dose which results in high likelihood of tumor control is not known for locally advanced NSCLCa, one can extrapolate from the dose–response data gained from early stage NSCLCa. Onishi et al. (2004) reported on the correlation between the BED and local tumor control using several SBRT dose and fractionation regime. They showed that at a BED of greater than 100 Gy results in a 92% likelihood of local control compared to 74% for BED less than 100 Gy. This resulted in a statistically significant improvement in 3 year overall survival of 88 vs. 69%, respectively.

In this study, we evaluate the dosimetric feasibility of chemoradiation therapy using standard fractionation to 50.4 Gy to control microscopic disease and systemic disease followed by an SBRT boost to gross tumor. We evaluated two SBRT dose levels, 16 and 28 Gy in two fractions. This study shows that at both the low and high dose levels, lung, heart, esophagus, and spinal cord dose can be kept low and within reasonable accepted dose constraints. The lung V20 and V5 for our cases was less than 30 and 55%, respectively. These are below the doses at which a high risk pulmonary toxicity is expected. Equally, the doses to other organs are within acceptable limits (Timmerman, 2008; Marks et al., 2010).

This study has potential limitations. The sample size is small. Although a variety of disease locations and distributions is represented by the evaluated patients (**Figure 1**). Not all possible disease distributions are represented. Thus, some patients who meet the inclusion criteria defined in Section “Methods” may not be able to meet the dose constraints set forth in this study. Furthermore, the dose constraints selected are based on commonly accepted dose constraints used for SBRT and conventional fractionation. Some of these dose constraints were established empirically or were based on conversions using the linear-quadratic model. This model although useful has limitations especially when using large dose per fraction. Thus, the dose constraints used in this study may or may not be optimal constraints. Nonetheless, the present study does support that at least some patients with stage III lung cancer can be treated with this approach and achieve reasonable dosimetric constraints to critical organs, and thus supports further clinical evaluation of this approach.

We therefore are planning a phase I dose escalation study to evaluate the clinical feasibility and any potential dose limiting toxicity of SBRT boost for stage III lung cancer. A starting dose of 16 Gy in two fractions will be used. The dose will then be increased by 2 Gy per fraction increments until a maximum dose of 28 Gy in two fractions or dose limiting toxicity is reached. **Table 4** shows the relative BED_10_ of each dose level. The initial dose level is roughly equivalent to the BED_10_ of a standard conventional fractionation treatment to 70 Gy. The dose escalation goal is to reach a BED_10_ of greater than 100 Gy. The maximum dose level was selected to be at a cumulative BED_10_ of less than 151 Gy, since toxicity for centrally located tumors has been reported at these doses (Timmerman et al., 2006). The maximum tolerated dose will then be evaluated for efficacy in a follow-up phase II trial.

**Table 4:** Biological equivalent dose (BED) and equivalent dose in 2 Gy per fraction (EQD2) for tumor control at each dose level.

Levels Dose	BED_10_	Cumulative^*^	BED10	EQD2	Cumulative^§^ EQD2
1	16Gy(8Gy x 2)	28.8	88.3	24	73.6
2	20Gy(10Gy x 2)	40	99.5	33.3	82.9
3	24Gy(12Gy x 2)	52.8	112.3	44	93.6
4	28Gy(14Gy x 2)	67.2	126.7	56	105.6

* Combine with 3D-CRT of 50.4 Gy (BED10 59.5).

§ Combine with 50.4 Gy 3D-CRT (EQD2 49.6).

## CONCLUSION

The combination of chemoradiation to 50.4 Gy followed by SBRT boost to gross disease at the primary tumor and involved regional lymph nodes is feasible with respect to normal tissue dose constraints in this dosimetric pilot study. A phase I/II trial to evaluate the clinical safety and efficacy of this approach is being undertaken.

## Conflict of Interest Statement

The authors declare that the research was conducted in the absence of any commercial or financial relationships that could be construed as a potential conflict of interest.
